# Red Algal Bromophenols as Glucose 6-Phosphate Dehydrogenase Inhibitors

**DOI:** 10.3390/md11104050

**Published:** 2013-10-22

**Authors:** Daisuke Mikami, Hideyuki Kurihara, Sang Moo Kim, Koretaro Takahashi

**Affiliations:** 1 Faculty and Graduate School of Fisheries Sciences, Hokkaido University, Minato, Hakodate, Hokkaido 041-8611, Japan; E-Mails: y-h.dmikami@fish.hokudai.ac.jp (D.M.); kore@fish.hokudai.ac.jp (K.T.); 2 Department of Marine Food Science and Technology, Gangneung-Wonju National University, Gangneung, Gangwon-do 210-702, Korea; E-Mail: smkim@gwnu.ac.kr

**Keywords:** bromophenol, Rhodomelaceae, pentose phosphate pathway (PPP), glucose 6-phosphate dehydrogenase, inhibition

## Abstract

Five bromophenols isolated from three Rhodomelaceae algae (*Laurencia nipponica*, *Polysiphonia morrowii*, *Odonthalia corymbifera*) showed inhibitory effects against glucose 6-phosphate dehydrogenase (G6PD). Among them, the symmetric bromophenol dimer (**5**) showed the highest inhibitory activity against G6PD.

## 1. Introduction

Marine algae are rich in secondary metabolites, such as terpenoids and polyphenols [1,2]. They contain many unique halogenated compounds, while terrestrial plants rarely contain them [3,4,5]. In particular, red algae of the family Rhodomelaceae contain a large amount of various bromophenols (about 1%–5% dry weight) [6,7]. Bromophenols show various beneficial functions, such as radical scavenging [8], anticancer [9], anti-inflammatory [10], antimicrobial [11] and α-glucosidase inhibitory activities [12]. 

Glucose 6-phosphate dehydrogenase (G6PD), the first key enzyme of pentose phosphate pathway (PPP), is mediated to generate reduced nicotinamide adenine dinucleotide phosphate (NADPH), a biological reductant of fatty acid and cholesterol biosynthesis in the lipogenic processes. Dehydroepiandrosterone (DHEA) is a well-known G6PD inhibitor [13] expected for as an antiobese agent [14]. Lipogenic activity and serum lipoprotein levels are decreased in G6PD-deficient patients, implying the importance of G6PD in fatty acid synthesis [15]. These observations indicate that G6PD is a potential therapeutic target for obesity. However it is problematic to use DHEA as an antiobese material. High oral administration of DHEA is required because it is easily converted to various active hormones [14]. G6PD is an important enzyme in tumor formation. Tumor cells require abundant lipids and nucleotides because of their rapid growth. Not only NADPH but also ribulose 5-phosphate (Ru5P), used for nucleotide synthesis, is produced in the PPP. Cell lines overexpressed G6PD caused formation of tumors in mice [16]. In contrast, G6PD-deficient tumor cell lines showed relatively slow growth and enhanced apoptosis [17]. There are only a few reports pertinent to G6PD inhibitors, steroids and its derivatives [18,19], and catechin gallates [20]. Thus, in the present study, we isolated bromophenols, as G6PD inhibitors, from marine red algae belonging to the Rhodomeraceae family.

## 2. Results and Discussion

**Figure 1:**
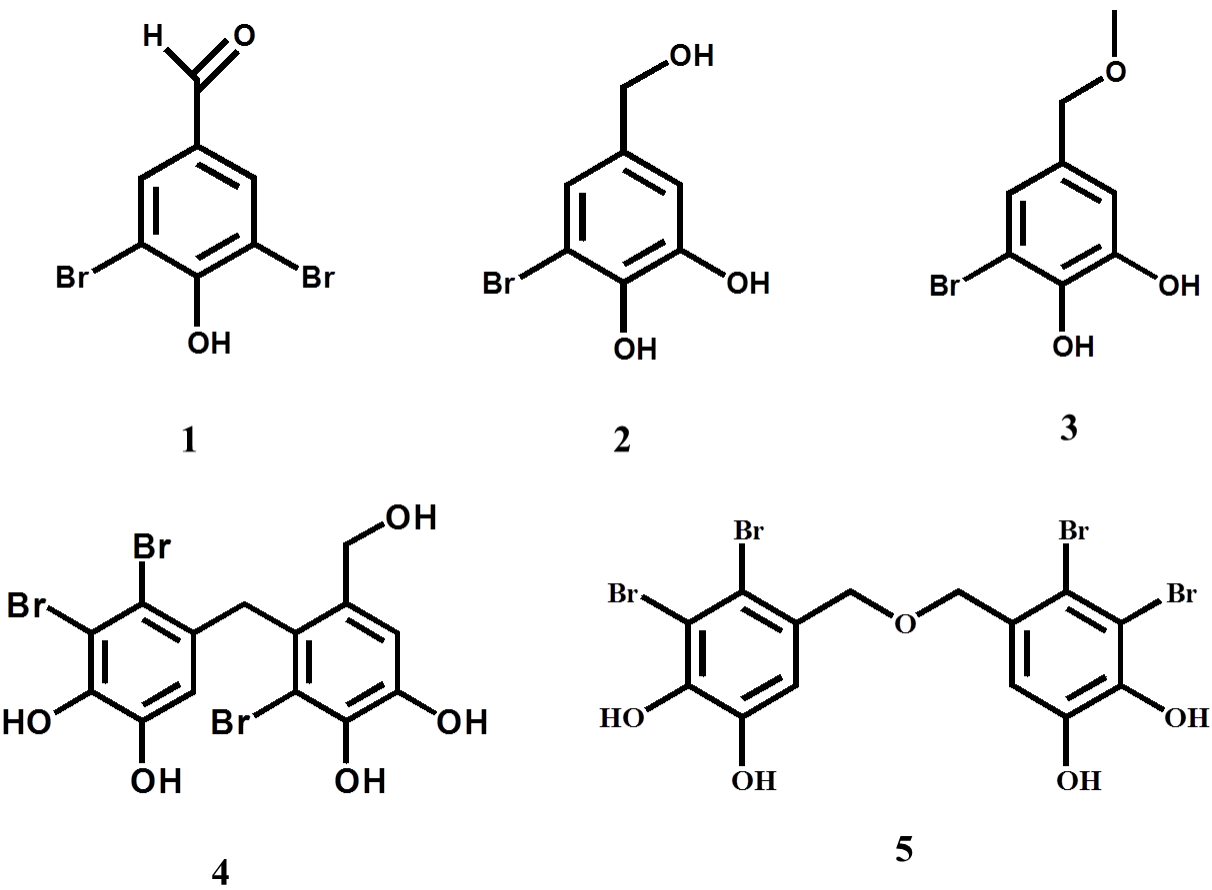
Bromophenols obtained from marine red algae in this study.

Assay-guided separation led to isolate five inhibitors **1**–**5** (Table 1) from marine red algae. Their structures were determined as shown in Figure 1, compared to the literature data [12,21,22,23]. Bromophenol dimers (**4**, **5**) were more effective inhibitors than their corresponding monomers (**1**, **2**, **3**). Similar relevance was observed in α-glucosidase inhibition [23]. In particular, the symmetric dibenzyl ether **5** was the most potent inhibitor (IC_50_ = 0.85 μM) among the bromophenols isolated. Bromophenol **5** showed 9.1-fold lower IC_50_ value than the known inhibitor epigallocatechin gallate (EGCG), while monobrominated phenols **2** and **3** showed low inhibition. The reason why bromophenol dimers show stronger inhibition than monomers is not understood. They would have relevance to Br atoms/molecule. Furthermore, inhibition may rely on nucleophilic substitution and/or spatial occupation in the active site of the enzyme. 

**Table 1:** IC_50_ of the bromophenols obtained and a positive control on glucose 6-phosphate dehydrogenase (G6PD) reaction.

Compound	IC_50_^a^ (μM)
**1**	76.6 ± 1.1
**2**	>370 (37.6 ± 1.6%) ^b^
**3**	>340 (35.7 ± 4.4%) ^c^
**4**	4.01 ± 0.30
**5**	0.85 ± 0.10
Epigallocatechin gallate	7.70 ± 0.14

^a^ Mean ± SD (*n =* 3). Inhibition assay was carried out at the substrate glucose 6-phosphate and NADP^+^ concentrations of 3.0 and 0.3 mM, respectively; ^b^ Inhibition (%) of compound **2** at the concentration of 370 μM; ^c^ Inhibition (%) of compound **3** at the concentration of 340 μM.

High substitution of Br atoms led to an increase in inhibitory potency against G6PD. Some researchers reported that highly brominated phenols showed stronger inhibition against enzymes [23,24]. This was considered to be the result of increased affinity to the enzymes due to debrominated aromatic nucleophilic substitution [23]. However, this is not sufficient to understand the relationship between the number of Br atoms and the enzyme inhibitory activity, because some highly brominated phenols showed similar enzyme inhibitory activities [25]. Bromophenol **2** showed identical inhibition with its corresponding methyl ether **3**. In the cases of α-glucosidase, the bromophenols with free alcoholic hydroxyl type significantly inhibited enzyme activities stronger than their methyl ethers [23].

This study is the first report on G6PD inhibitors obtained from marine red algae. Compound **5** was also found in the edible alga *Polyopes lancifolia* as a stable compound [26]. In addition, a previous study described compound **5** as a weak inhibitior (IC_50_ = 1.0–1.2 mM) for purified α-glucosidase [26]. This suggests that compound **5** is not a nonspecific inhibitor, whereas most polyphenolics nonspecifically interact with proteins. These bromophenol containing algae or bromophenol are expected to be utilized for food stuffs or neutraceuticals, although further study would be required to desclose cytotoxity and metabolic behavior *in vivo*.

## 3. Experimental Section

### 3.1. General

G6PD (EC 1.1.1.49) from *Leuconostoc mesenteroides* was purchased from Sigma-Aldrich (St. Louis, MO, USA). WST-1 and 1-methoxy-5-methylphenazinium methylsulfate (1-methoxy PMS) were purchased from Dojindo Laboratories (Mashiki, Kumamoto, Japan) and oxidized nicotinamide adenine dinucleotide phosphate (NADP^+^) from Oriental Yeast Industries (Tokyo, Japan). Glucose 6-phosphate was purchased from Wako Pure Chemicals (Tokyo, Japan). Epigallocatechin gallate (EGCG) was purchased from Cayman Chemical Company (Ann Arbor, MI, USA). Thin layer chromatography (TLC) was carried out using a glass plate precoated with silica gel 60 F_254_ and RP-18 (Merck, Darmstadt, Germany), and spots were detected under UV light and visualized by spraying 50% sulfuric acid and potassium ferricyanide-ferric chloride reagents. NMR spectra were recorded in acetone-*d*_6_ on a Bruker AMX-500 (Karlsruhe, Germany) spectrometer at 500 MHz for proton and 125 MHz for carbon chemical shifts. Field desorption-MS spectra were recorded on a JEOL JMS-T100GCV spectrometer (Tokyo, Japan).

### 3.2. Algal Material

The algae *Laurencia nipponica*, *Odonthalia corymbifera* and *Polysiphonia morrowii* were collected at Nemuro, Muroran and Hakodate in Hokkaido, Japan, respectively, in 2010–2012. They were identified by Hajime Yasui, Faculity of Fisheries sciences, Hokkaido University. The alga *L. nipponica* was stored as frozen sample. The algae *O. corymbifera* and *P. morrowii* were immediately brought to our laboratory and then extracted according to the following experiments described.

### 3.3. Enzyme Assay

Enzyme assay was carried out by colorimetric method as described in literature with slight modification [27]. The reaction mixture was prepared by adding 135 mM Tris-HCl buffer (pH 7.8, 675 μL), 30 mM glucose 6-phosphate (100 μL), 3 mM NADP^+^ (100 μL), 20 mM MgCl_2_ (100 μL) and test materials in MeOH (15 μL). Reaction was initiated by adding 0.035 U/mL G6PD solution (10 μL) to the reaction mixture. Each reaction was carried out at 25 °C for 15 min and terminated by adding 1 mL of saturated aqueous NaCl solution. For determination of produced NADPH, 0.05 mM WST-1 (400 μL) and 0.025 mM 1-methoxy PMS (400 μL) were mixed to the reaction mixture (400 μL) and the absorbance was measured at 438 nm. EGCG was used as a positive control [20].

### 3.4. Extraction and Purification of G6PD Inhibitors

Collected algae were washed with tapped water, then cut into small pieces, and soaked in 95% aqueous acetone for *L. nipponica* or MeOH for *O. corymbifera*, *P. morrowii*, for 2 to 3 days. Organic solvent was evaporated under reduced pressure. Then, the residues were suspended in water and successively extracted with *n*-hexane, EtOAc and *n*-butanol. With the guidance of inhibition assay, G6PD inhibitors were separated by a combination of several chromatographic methods. *L. nipponica* EtOAc-soluble fraction (2.478 g, 75.6% inhibition at 100 μg/mL) was chromatographed on silica gel (Wakogel C-100, Wako Pure Chemicals) to obtain the inhibitory fraction (780 mg, 28.0% inhibition at 40 μg/mL) eluted with toluene/EtOAc = 9:1 (v/v). The fraction was further purified by preparative silica gel TLC developed with toluene/EtOAc/acetone = 6:1:1 (v/v/v). Final purification was done by silica gel HPLC (ULTRON VX-SIL, Shinwa Chemical Industries, *n*-Hexane/EtOH/AcOH = 10:1:0.055 (v/v/v)) to isolate compound **1** (4.2 mg, 0.00033% of air-dried weight). *P. morrowii* EtOAc-soluble fraction (1.987 g, 21.2% inhibition at 50 μg/mL) was chromatographed on silica gel to afford two inhibitory fractions A (311 mg, 27.8% inhibition at 20 μg/mL) eluted with toluene/EtOAc = 8:2 (v/v) and B (144 mg, 34.8% inhibition at 20 μg/mL) eluted with toluene/EtOAc = 2:8 (v/v). Fraction A was further purified by octa decyl silyl (ODS) column chromatography (Cosmosil 140C_18_-OPN, Nacalai tesque) eluted with 40% aqueous acetone, and ODS HPLC (Mightysil RP-18, Kanto Chemical, Tokyo, Japan) to obtain compound **3** (118 mg, 0.0219% of air-dried weight), eluted with 20% aqueous acetonitrile. Fraction B was purified by ODS column chromatography eluted with 30% aqueous acetone, and ODS HPLC to obtain compound **2** (15.5 mg, 0.00287% of air-dried weight), eluted with 40% aqueous MeOH. *O. corymbifera* EtOAc-soluble fraction (4.608 g, 25.5% inhibition at 10 μg/mL) was chromatographed on silica gel to afford two inhibitory fractions C (1204 mg, 31.7% inhibition at 5 μg/mL) eluted with toluene/EtOAc = 6:4 (v/v) and D (557 mg, 38.9% inhibition at 5 μg/mL) eluted with toluene/EtOAc = 2:8 (v/v). Fraction C was further purified by ODS column chromatography to obtain compound **5** (174 mg, 0.0348% of air-dried weight) eluted with 60% aqueous MeOH. Fraction D was further purified by ODS column chromatography eluted with 50% aqueous acetone, and ODS HPLC to obtain compound **4** (10.0 mg, 0.00100% of air-dried weight) eluted with 60% aqueous MeOH. 

The MS and NMR data of compounds **1**–**5** are listed as follows (see Supplementary Information for HPLC chromatogram and NMR spectra):

Compound **1**, ^1^H-NMR (500 MHz, acetone-*d*_6_): δ 9.84 (s, 1H, –CHO), 8.08 (s, 2H, H-2); ^13^C-NMR (125 MHz, acetone-*d*_6_): δ 89.24 (–CHO), 156.77 (C-4), 134.52 (C-2), 132.11 (C-1), 112.02; EI-MS: *m*/*z* 277 [M − H]^+^ (68), 278 [M]^+^ (15), 279 [M − H + 2]^+^ (100), 280 [M + 2]^+^ (23), 281 [M − H + 4]^+^ (55), 282 [M + 4]^+^ (11); EI-HR-MS: *m*/*z* 276.8469 [M − H]^+^ (calculated 276.8500 for C_7_H_3_O_2_^79^Br_2_).

Compound **2**, ^1^H-NMR (500 MHz, acetone-*d*_6_): δ 6.95 (d, *J* = 1.68, 1H, H-2), 6.84 (d, *J* = 1.68, 1H, H-6), 4.45 (s, 2H, H-7); ^13^C-NMR (125 MHz, acetone-*d*_6_): δ 146.54 (C-5), 142.53 (C-4), 136.17 (C-1), 122.35 (C-2), 113.88 (C-6), 109.79 (C-3), 63.83 (C-7); EI-MS: *m*/*z* 218 [M]^+^ (100), 220 [M + 2]^+^ (98); EI-HR-MS: *m*/*z* 217.9599 [M]^+^ (calculated 217.9579 for C_7_H_7_O_3_^79^Br).

Compound **3**, ^1^H-NMR (500 MHz, acetone-*d*_6_): δ 6.95 (d, *J* = 1.89, 1H, H-2), 6.82 (d, *J* = 1.89, 1H, H-6), 4.26 (s, 2H, H-7), 3.26 (s, 3H, –OCH_3_); ^13^C-NMR (125 MHz, acetone-*d*_6_): δ 146.47 (C-5), 143.00 (C-4), 132.31 (C-1), 123.48 (C-2), 114.71 (C-6), 109.72 (C-3), 73.98 (C-7), 57.76; EI-MS: *m*/*z* 232 [M]^+^ (79), 234 [M + 2]^+^ (75); EI-HR-MS: *m*/*z* 231.9758 [M]^+^ (calculated 231.9736 for C_8_H_9_O_3_^79^Br).

Compound **4**, ^1^H-NMR (500 MHz, acetone-*d*_6_): δ 7.08 (s, 1H, H-6), 6.07 (s, 1H, H-6′), 4.40 (s, 2H, H-8), 4.11 (s, 2H, H-7); ^13^C-NMR (125 MHz, acetone-*d*_6_): δ 145.44 (C-5′), 145.00 (C-5), 143.58 (C-4′), 142.82 (C-4), 134.33 (C-2), 132.27 (C-1), 128.23 (C-1′), 116.27 (C-3), 115.06 (C-6), 114.82 (C-6′), 114.74 (C-2′), 113.62 (C-3′), 62.63 (C-8), 39.28 (C-7); FD-MS: *m*/*z* 496 [M]^+^ (37), 498 [M + 2]^+^ (100), 500 [M + 4]^+^ (95), 502 [M + 6]^+^ (33); FD-HR-MS: *m*/*z* 495.8183 [M] ^+^ (calculated 495.8157 for C_14_H_11_O_5_^79^Br_3_).

Compound **5**, ^1^H-NMR (500 MHz, acetone-*d*_6_): δ 7.14 (s, 2H, H-6), 4.60 (s, 4H, H-7); ^13^C-NMR (125 MHz, acetone-*d*_6_): δ 145.63 (C-4 or C5), 144.73 (C-4 or C5), 131.28 (C-1), 115.64 (C-6), 114.74 (C-2 or C3), 113.77 (C-2 or C-3), 73.31; FD-MS: *m*/*z* 574 [M]^+^ (25), 576 [M + 2]^+^ (87), 578 [M + 4]^+^ (100), 580 [M + 6]^+^ (85), 582 [M + 8]^+^ (24); FD-HR-MS: *m*/*z* 573.7239 [M]^+^ (calculated 573.7262 for C_14_H_10_O_5_^79^Br_4_).

## 4. Conclusions

Rhodomeraceae algae are rich sources of G6PD inhibitors. The inhibitors were identified as bromophenols (**1**–**5**). The symmetric bromophenol dimer **5** was the most potent inhibitor among them.

## Supplementary Files

Supplementary File 1 Supplementary Materials (PDF, 469 KB)
