# Supplementary material for: Red Algal Bromophenols as Glucose 6-Phosphate Dehydrogenase Inhibitors
Source: Mar Drugs. 2013 Oct 22;11(10):4050–7. doi: 10.3390/md11104050 (PMC3826149; doi:10.3390/md11104050)

Supplementary Materials

Figure S1. HPLC chromatogram of **1**...2
Figure S2. ^{1}H-NMR spectrum of **1** in acetone-d_6 at 500 MHz...3
Figure S3. ^{13}C-NMR spectrum of **1** in acetone-d_6 at 125 MHz...4
Figure S4. HPLC chromatogram of **2**...5
Figure S5. ^{1}H-NMR spectrum of **2** in acetone-d_6 at 500 MHz...6
Figure S6. ^{13}C-NMR spectrum of **2** in acetone-d_6 at 125 MHz...7
Figure S7. HPLC chromatogram of **3**...8
Figure S8. ^{1}H-NMR spectrum of **3** in acetone-d_6 at 500 MHz...9
Figure S9. ^{13}C-NMR spectrum of **3** in acetone-d_6 at 125 MHz...10
Figure S10. HPLC chromatogram of **4**...11
Figure S11. ^{1}H-NMR spectrum of **4** in acetone-d_6 at 500 MHz...12
Figure S12. ^{13}C-NMR spectrum of **4** in acetone-d_6 at 125 MHz...13
Figure S13. HPLC chromatogram of **5**...14
Figure S14. ^{1}H-NMR spectrum of **5** in acetone-d_6 at 500 MHz...15
Figure S15. ^{13}C-NMR spectrum of **5** in acetone-d_6 at 125 MHz...16

Figure S1. HPLC chromatogram of compound **1**.

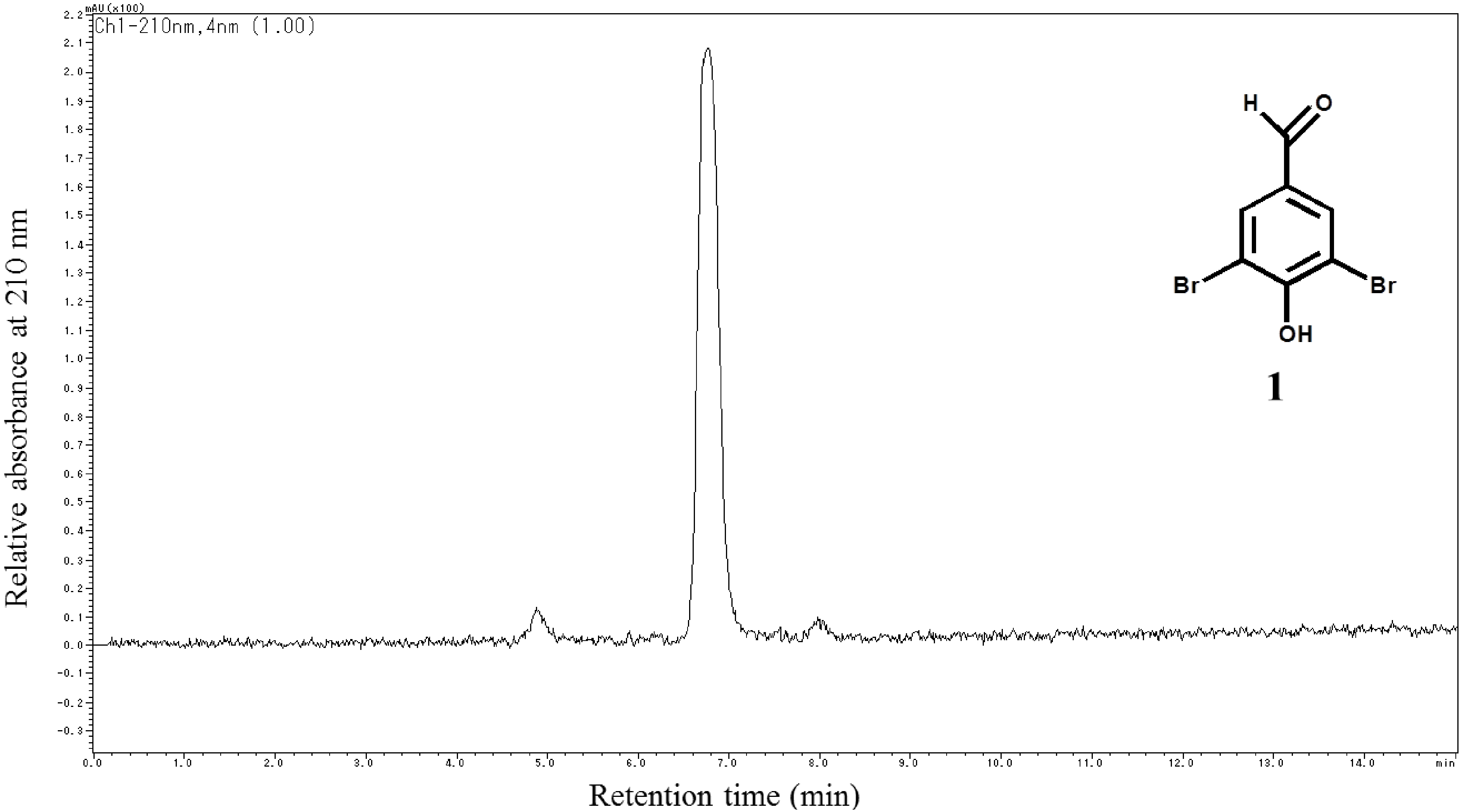

HPLC conditions: Columun: ULTRON VX-SIL RP-18 (φ 4.6 ×250 mm); Mobile phase: *n*-Hexane/EtOH/AcOH = 10:1:0.05 (v/v/v); Flow rate: 0.8 mL/min; Detection: UV 210 nm.

Figure S2. ^{1}H-NMR spectrum of compound **1** in acetone-d_6 at 500 MHz.

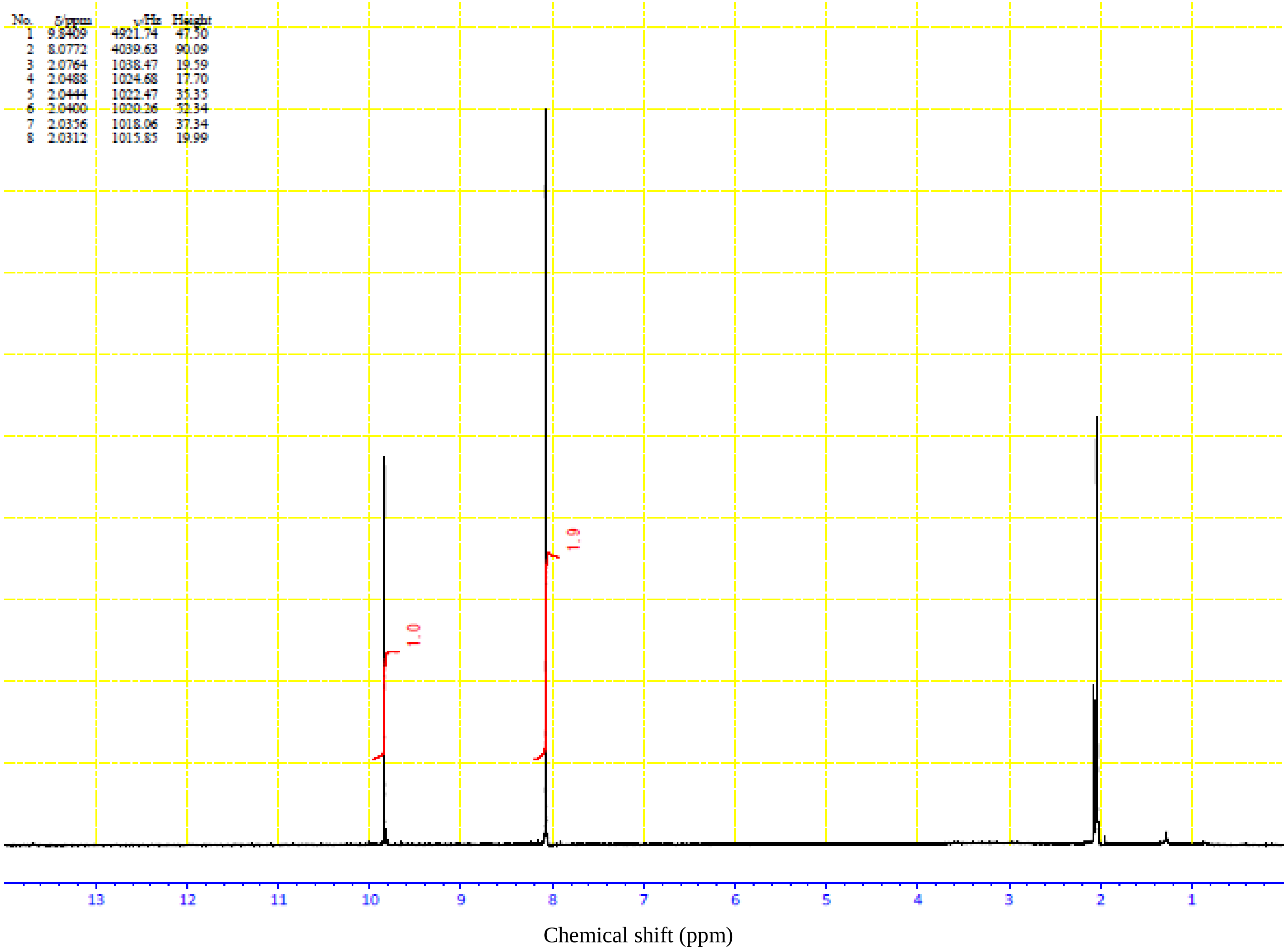

Figure S3. ^{13}C-NMR spectrum of compound **1** in acetone-d_6 at 125 MHz.

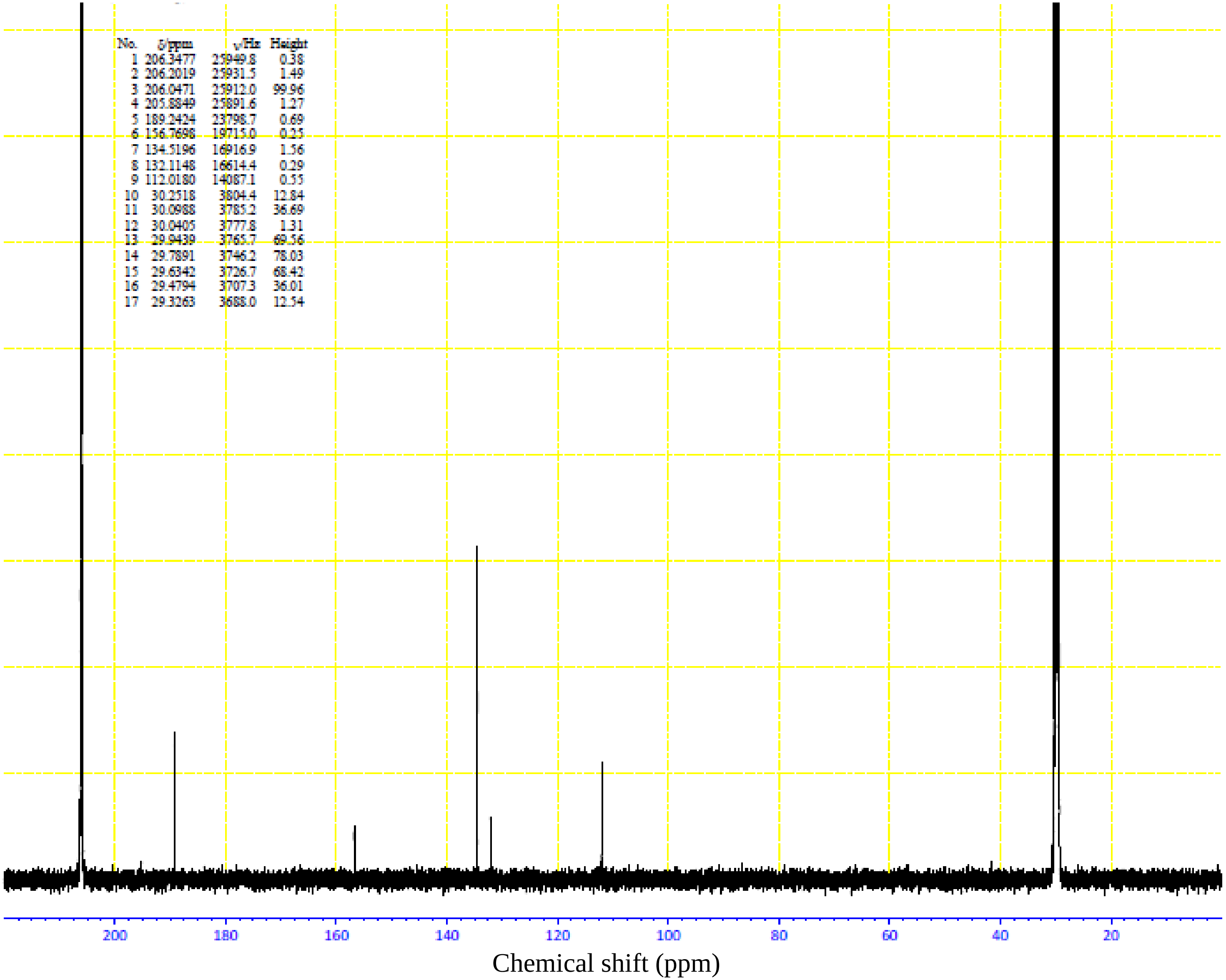

Figure S4. HPLC chromatogram of compound **2**.

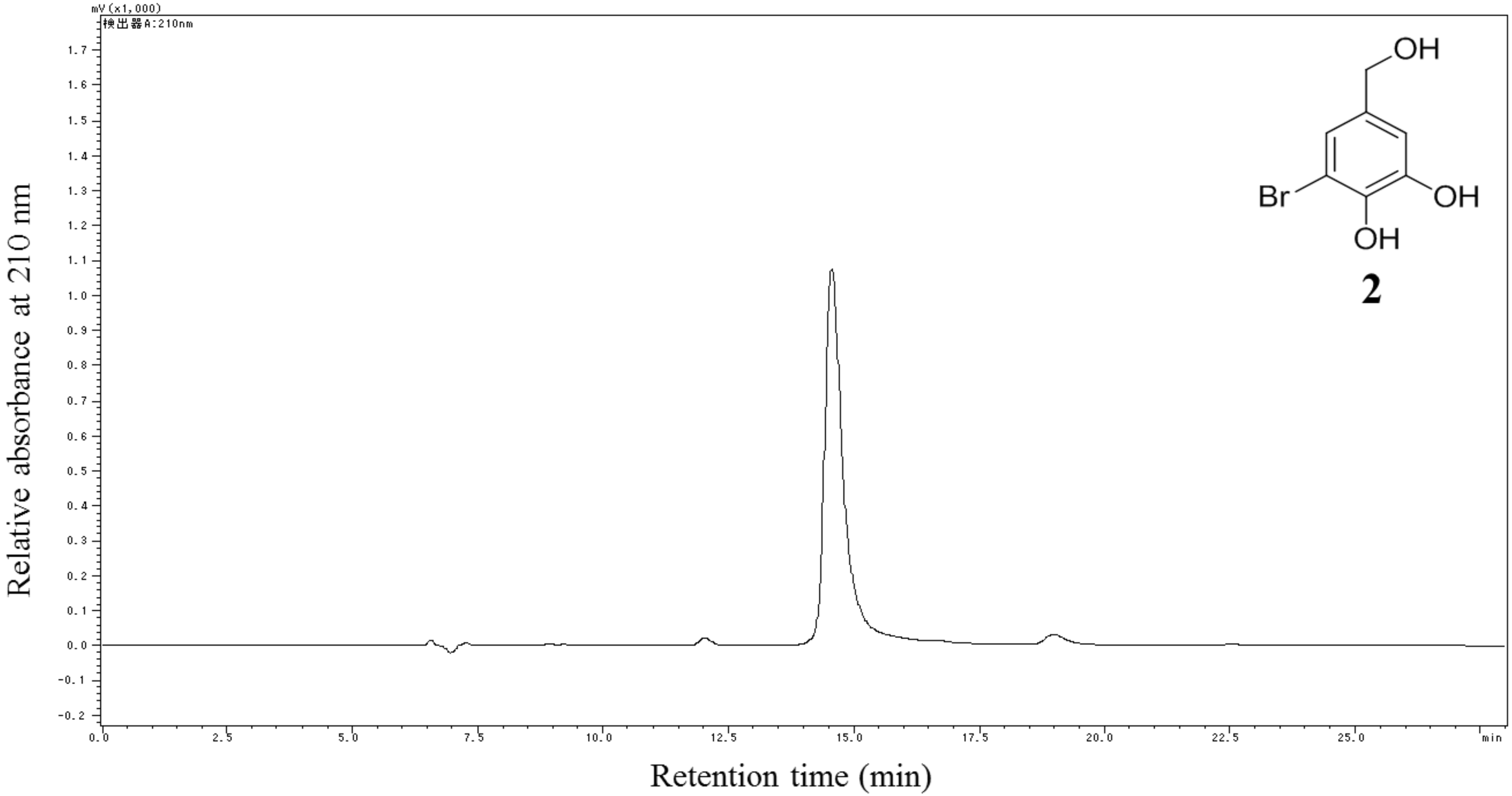

HPLC conditions: Columun: Mightysil RP-18 250–4.6 (5 μm); Mobile phase: 20% aqueous CH_3CN; Flow rate: 0.5 mL/min; Detection: UV 210 nm.

Figure S5. ^{1}H-NMR spectrum of **2** in acetone-d_6 at 500 MHz.

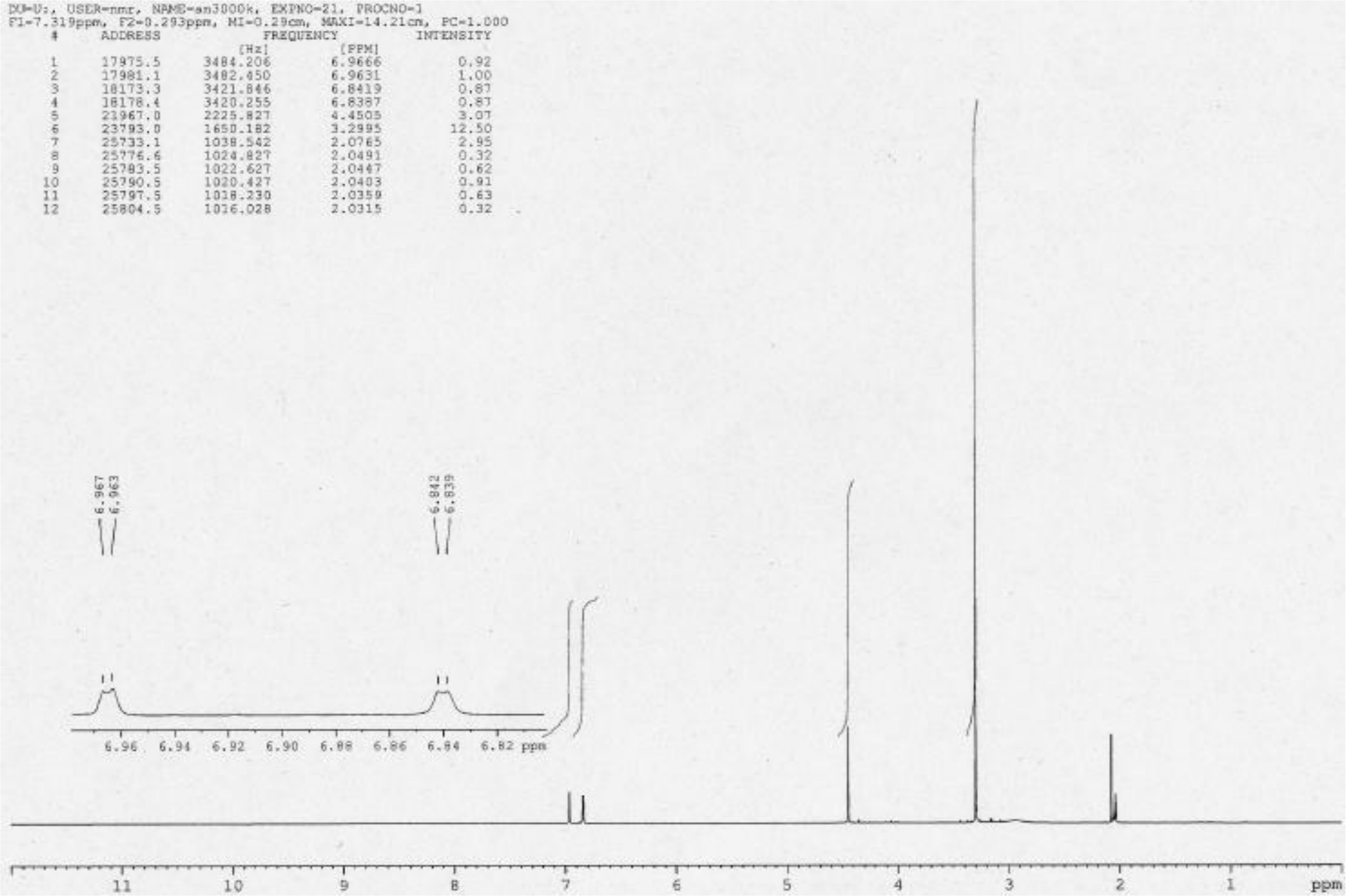

Chemical shift (ppm)

Figure S6. ^{13}C-NMR spectrum of **2** in acetone-d_6 at 125 MHz.

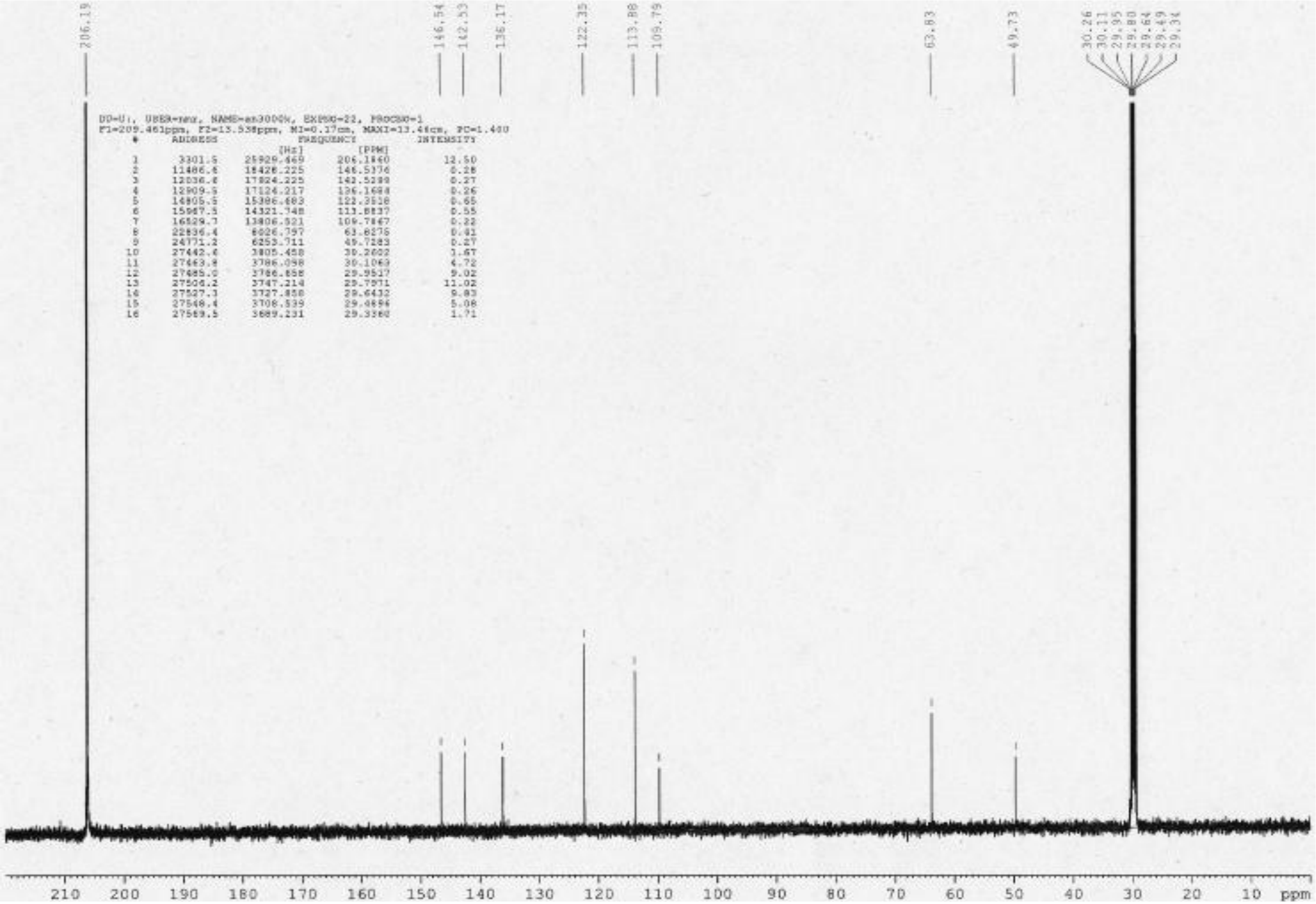

Chemical shift (ppm)

Figure S7. HPLC chromatogram of **3**.

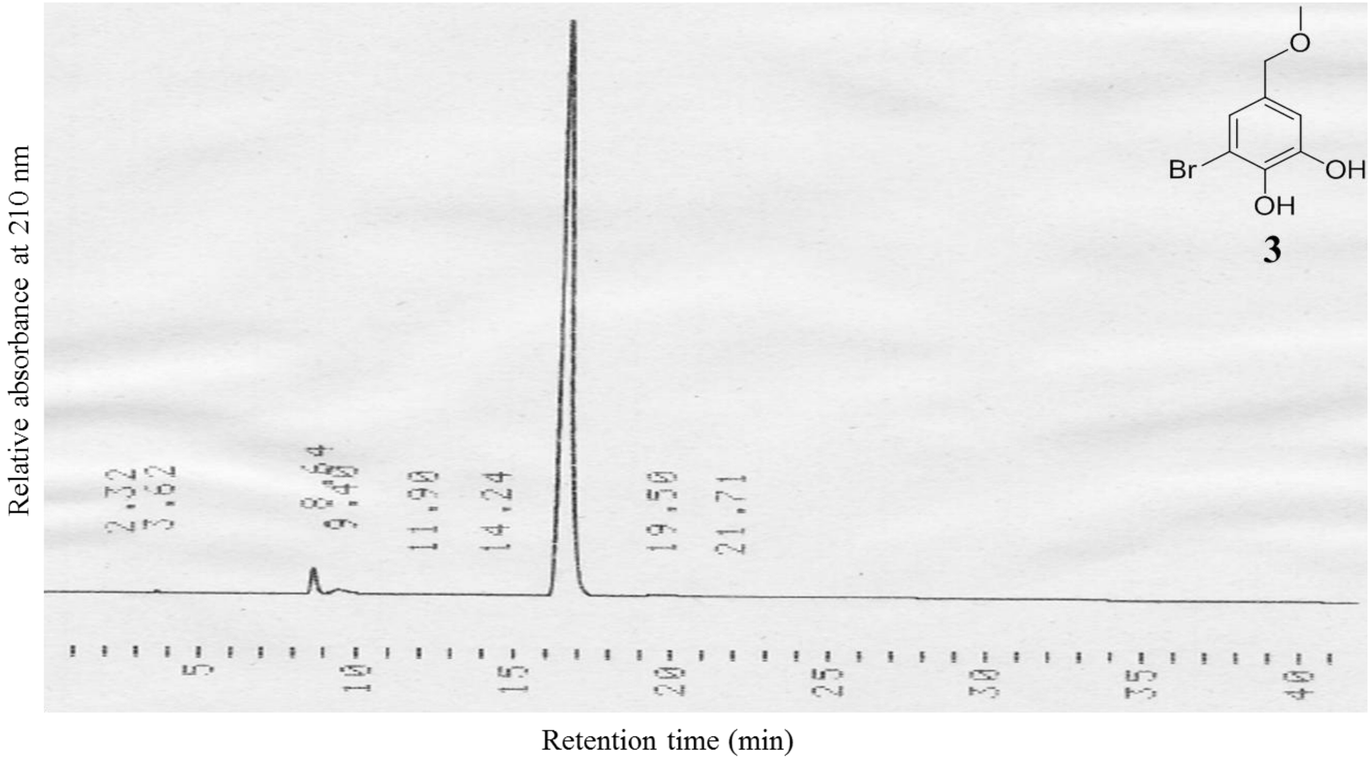

HPLC conditions: Columun: Mightysil RP-18 250–4.6 (5 μm); Mobile phase: 40% aqueous MeOH; Flow rate: 0.8 mL/min; Detection: UV 210 nm.

Figure S8. ^{1}H-NMR spectrum of **3** in acetone-d_6 at 500 MHz.

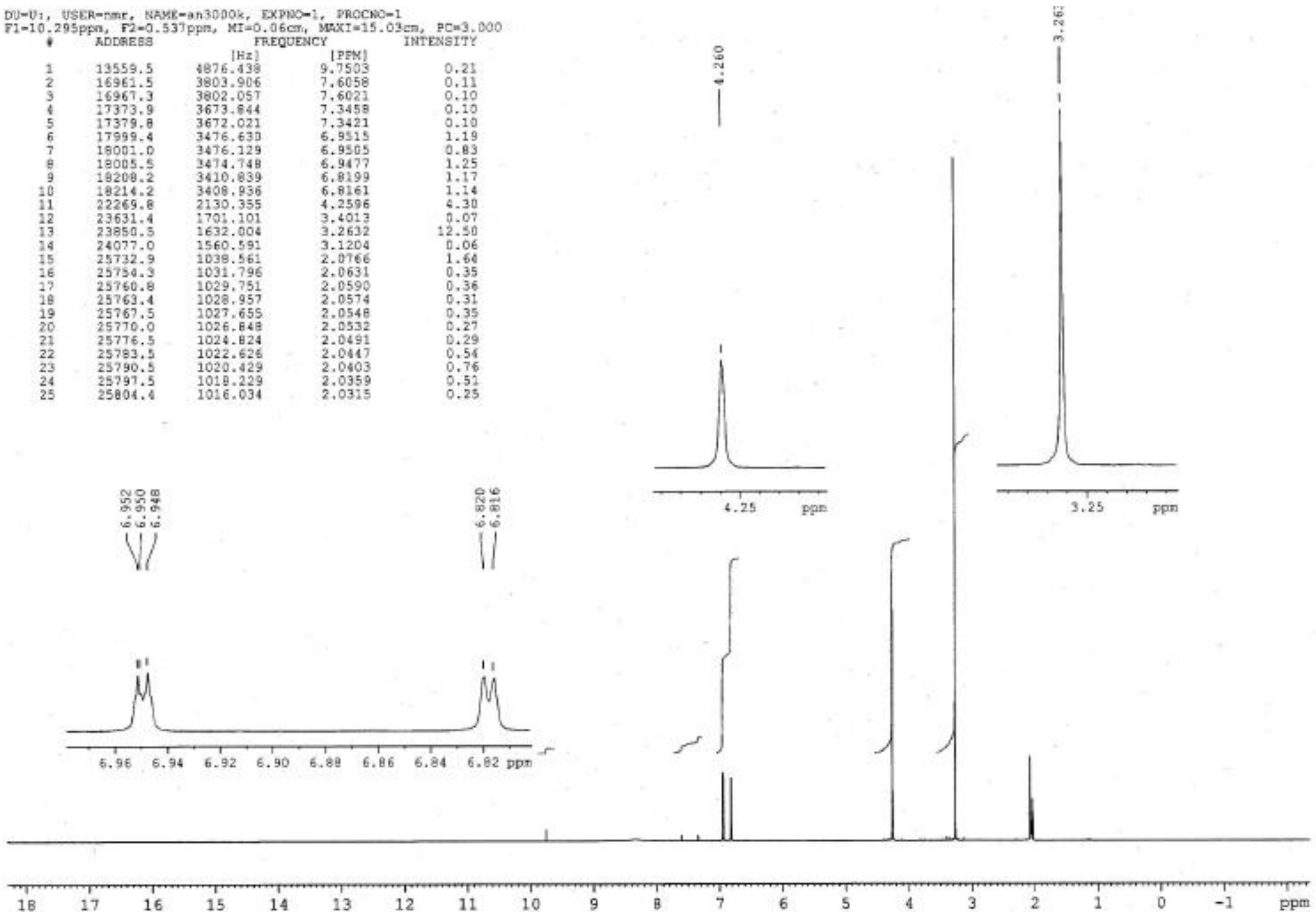

Chemical shift (ppm)

Figure S9. ^{13}C-NMR spectrum of **3** in acetone-d_6 at 125 MHz.

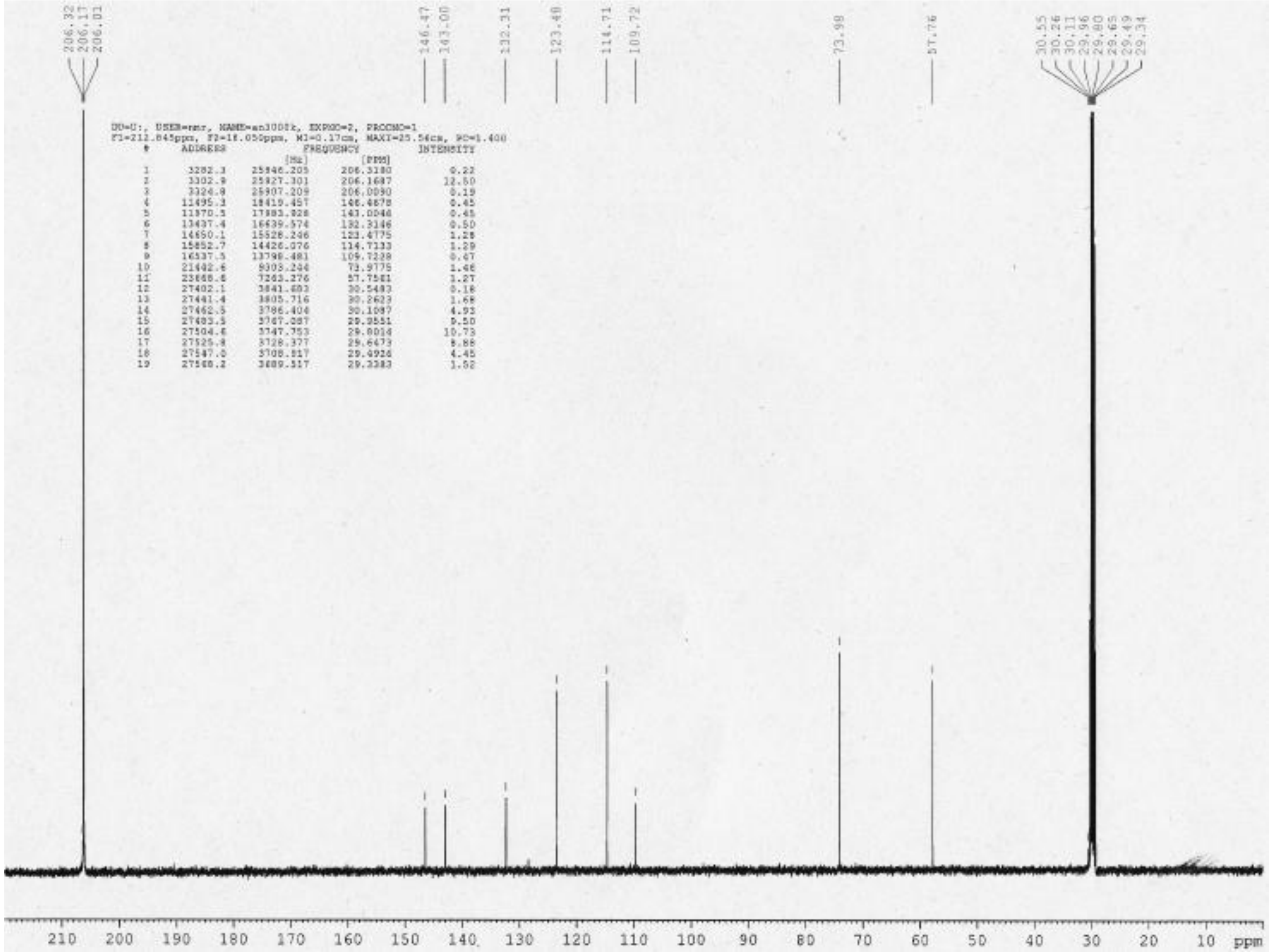

Chemical shift (ppm)

Figure S10. HPLC chromatogram of **4**.

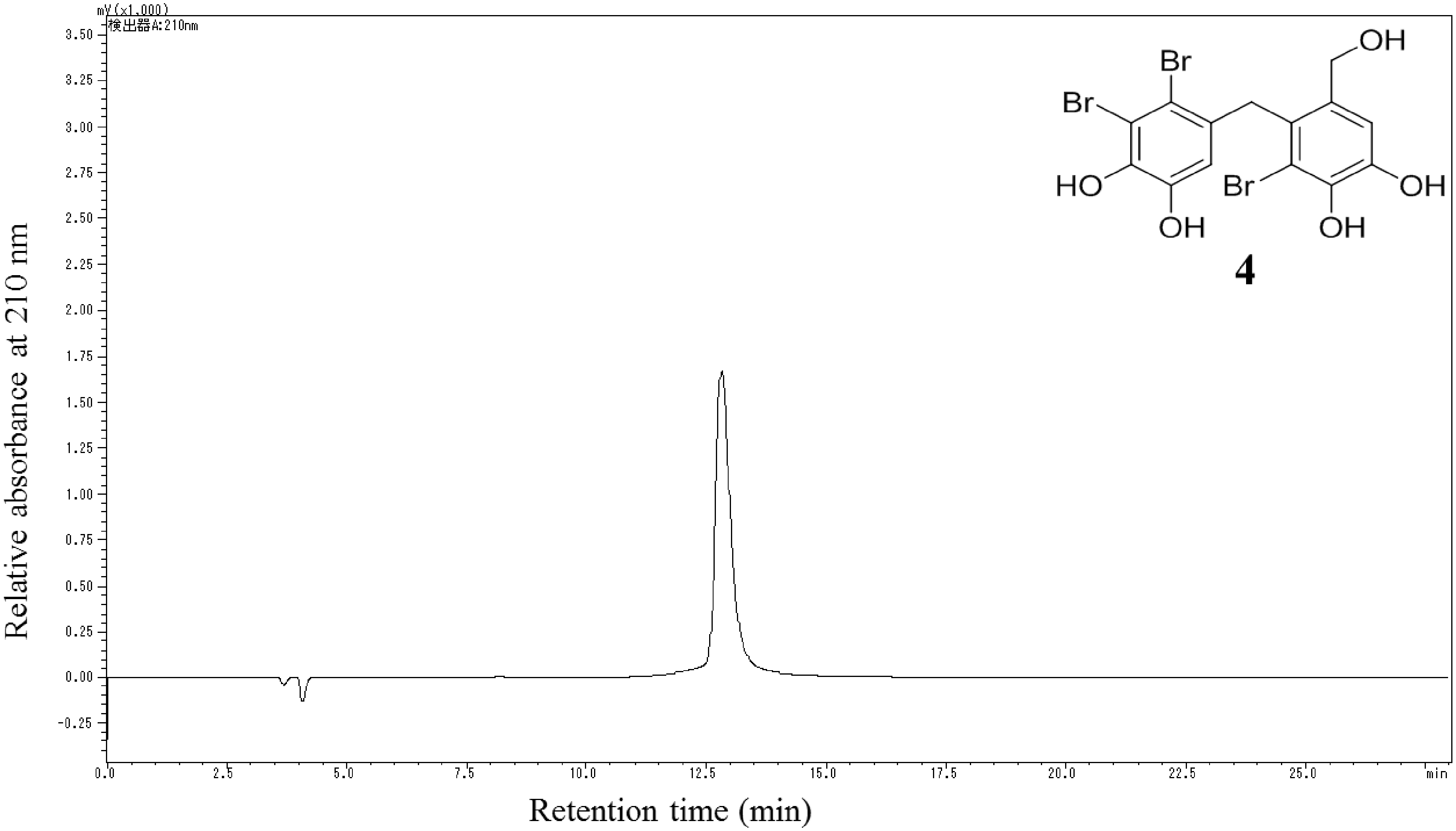

HPLC conditions: Columun: Mightysil RP-18 250–4.6 (5 µm); Mobile phase: 60% aqueous MeOH + 0.1% AcOH; Flow rate: 0.8 mL/min; Detection: UV 210 nm.

Figure S11. ^{1}H-NMR spectrum of **4** in acetone-d_6 at 500 MHz.

#	ADDRESS	FREQUENCY [Hz]	[PPM]	INTENSITY
1	17790.7	3542.463	7.0831	0.32
2	19394.3	3036.915	6.0723	0.61
3	22053.2	2198.682	4.3962	1.16
4	22502.1	2057.138	4.1132	1.33
5	25732.8	1038.609	2.0767	12.50
6	25754.4	1031.801	2.0631	0.58
7	25760.9	1029.769	2.0590	0.55
8	25767.6	1027.673	2.0548	0.53
9	25776.6	1024.806	2.0491	0.96
10	25783.6	1022.614	2.0447	1.73
11	25790.6	1020.425	2.0403	2.42
12	25797.5	1018.230	2.0359	1.72
13	25804.4	1016.041	2.0316	0.90
14	27144.2	593.635	1.1870	0.27

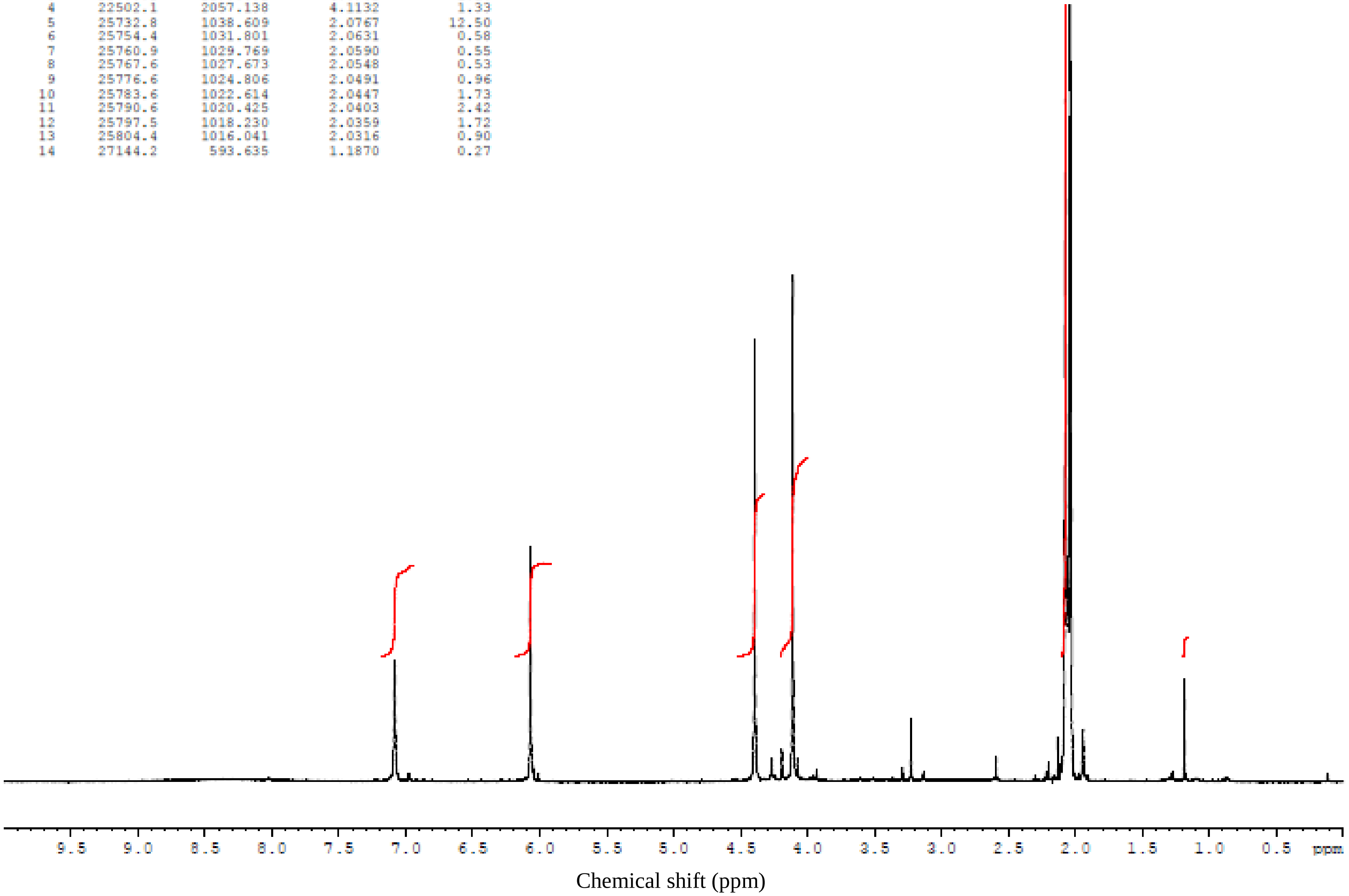

Figure S12. ^{13}C-NMR spectrum of **4** in acetone-d_6 at 125 MHz.

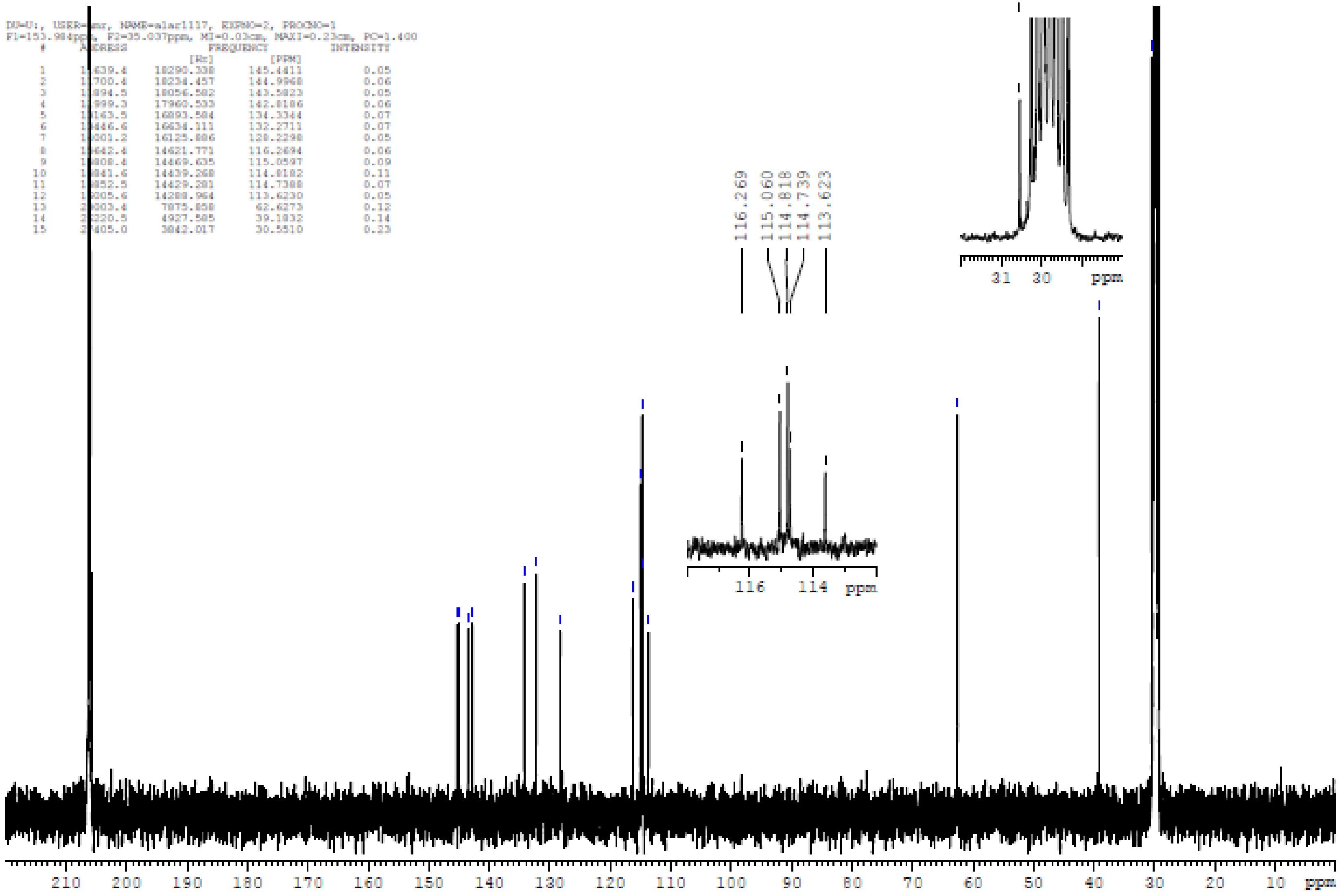

Chemical shift (ppm)

Figure S13. HPLC chromatogram of **5**.

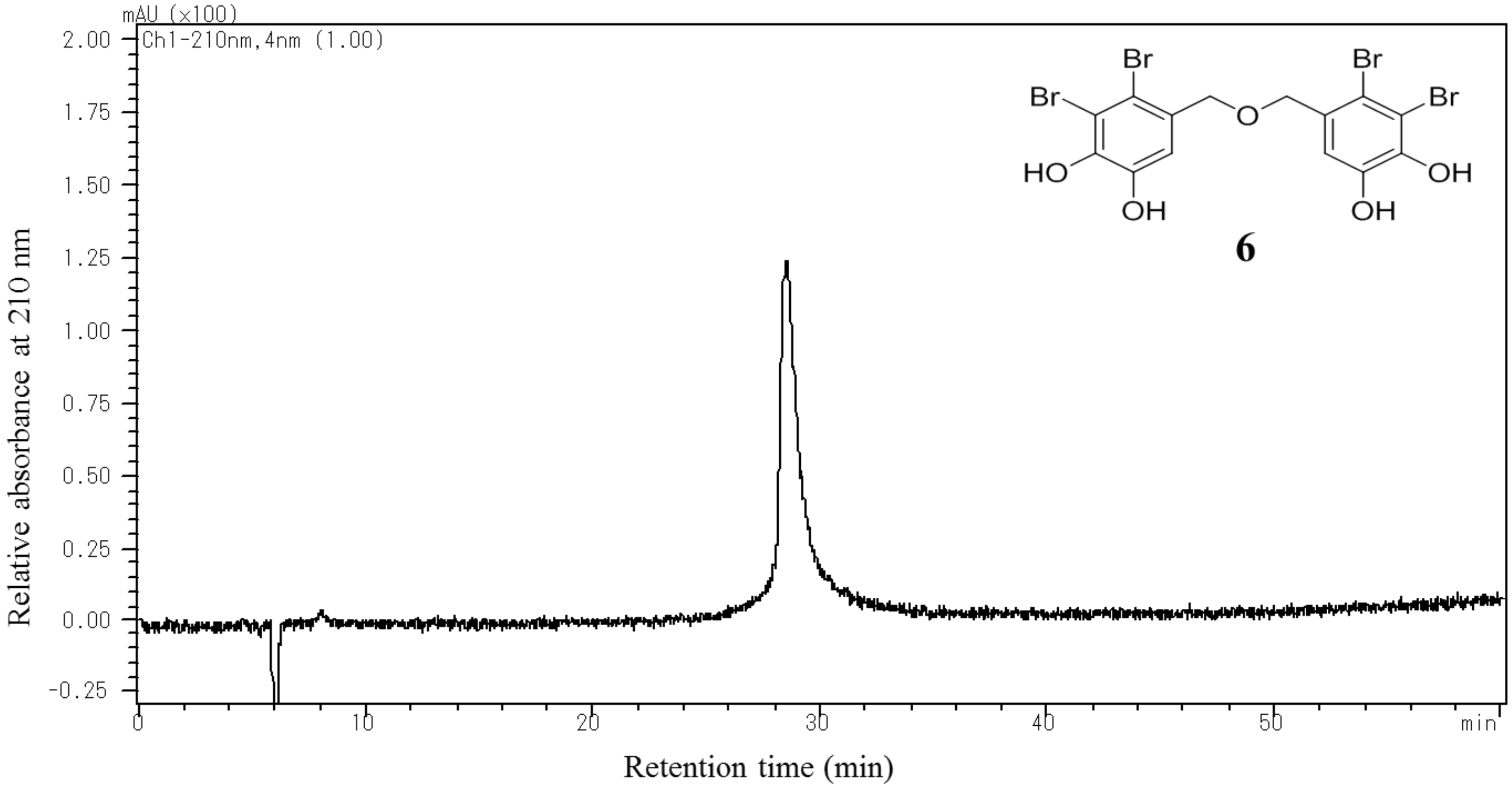

HPLC conditions: Columun: Mightysil RP-18 250–4.6 (5 μm); Mobile phase: 50% aqueous CH_3CN + 0.1% AcOH; Flow rate: 0.5 mL/min; Detection: UV 210 nm.

Figure S14. ^{1}H-NMR spectrum of **5** in acetone-d_6 at 500 MHz.

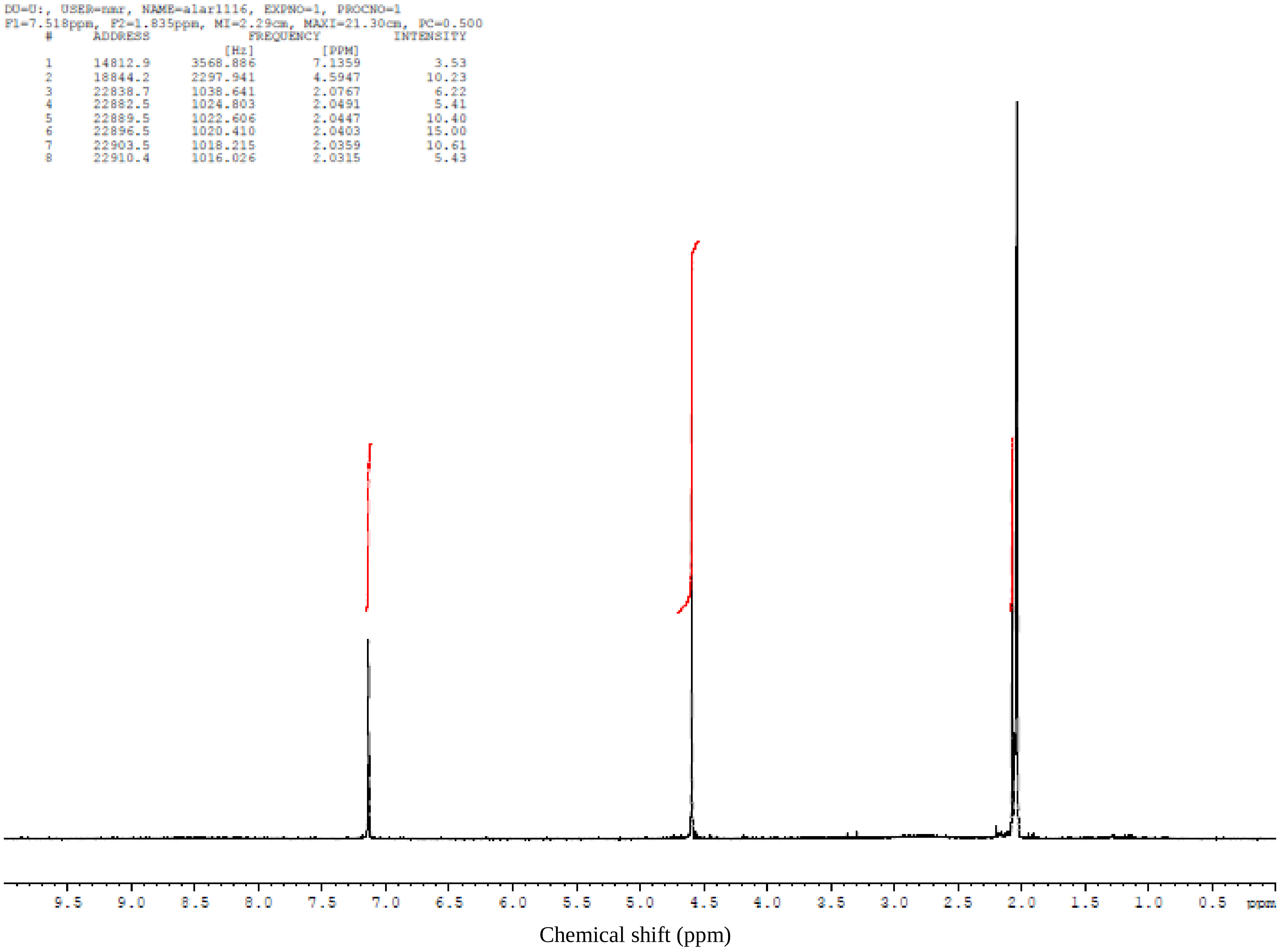

Figure S15. ^{13}C-NMR spectrum of **5** in acetone-d_6 at 125 MHz.

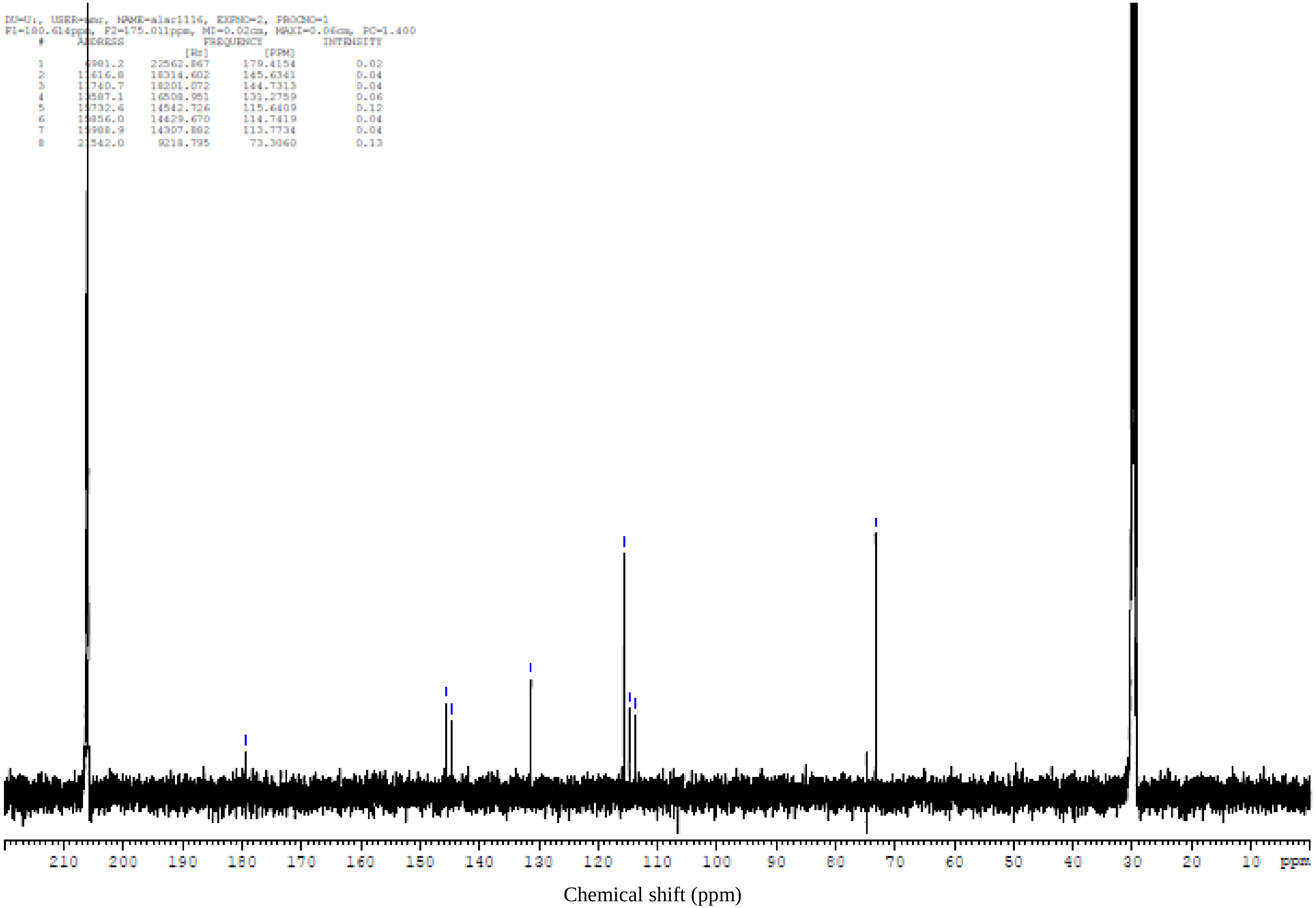

Supplement: Supplementary File 1 — Supplementary Materials (PDF, 469 KB) [file marinedrugs-11-04050-s001.pdf]
